# The Role of RPGR and Its Interacting Proteins in Ciliopathies

**DOI:** 10.1155/2015/414781

**Published:** 2015-06-01

**Authors:** Sarita Rani Patnaik, Rakesh Kotapati Raghupathy, Xun Zhang, David Mansfield, Xinhua Shu

**Affiliations:** ^1^Department of Life Sciences, Glasgow Caledonian University, Glasgow G4 0BA, UK; ^2^Inverclyde Royal Hospital, Greenock PA16 0XN, UK

## Abstract

Ciliopathies encompass a group of genetic disorders characterized by defects in the formation, maintenance, or function of cilia. Retinitis pigmentosa (RP) is frequently one of the clinical features presented in diverse ciliopathies. RP is a heterogeneous group of inherited retinal disorders, characterized by the death of photoreceptors and affecting more than one million individuals worldwide. The *retinitis pigmentosa GTPase regulator* (*RPGR*) gene is mutated in up to 20% of all RP patients. RPGR protein has different interacting partners to function in ciliary protein trafficking. In this review, we specifically focus on RPGR and its two interacting proteins: RPGRIP1 and RPGRIP1L. We summarize the function of the three proteins and highlight recent studies that provide insight into the cellular function of those proteins.

## 1. Introduction: Cilia and Photoreceptor

### 1.1. Cilia Architecture

Cilia are tiny, hair-like structures protruding from the cell surface. They are highly conserved organelles and serve a variety of sensory functions in both unicellular and multicellular organisms. Historically, cilia have been classified into two categories: motile and nonmotile cilia (primary cilia), depending on the arrangement of the microtubule triplets, termed the axoneme. The axoneme of motile cilia is built in the classical 9+2 arrangement, where nine outer microtubule doublets surround a central pair of singlet microtubules. The axoneme of primary cilia lacks the central pairs of microtubules and is arrayed in a 9+0 configuration [1]. Primary cilia are immotile and can sense extracellular physical and biochemical signals, acting as a coordination centre of multiple signal transduction pathways [2]. Some epithelial cell surfaces contain a large number of motile cilia, which beat cooperatively to generate fluid movement. For instance, cells lining in the epididymis, the oviducts, the respiratory tract, and ependymal surfaces of the brain have large clusters of motile cilia, which beat in coordinated waves and perform a broad range of functions. The central microtubules within the motile cilia help in the bending motion. Flagella are found on single-celled eukaryotes and sperm cells and are primarily involved in cell locomotion. Prokaryotic flagella are structurally similar to eukaryotic flagella, although there are distinctions made according to the function and the length. Flagella also have a 9+2 axoneme arrangement which is similar to that of motile cilia.

### 1.2. Protein Transport in Cilia

All protein synthesizing machineries are restricted to the cytoplasm, so continued elongation of the cilium requires the selective import and transport of ciliary proteins by intraflagellar transport (IFT), which mediates bidirectional movement of multiprotein loaded particles along the axoneme. IFT was initially discovered in flagella of* Chlamydomonas reinhardtii*, in which anterograde and retrograde movements of particles were observed through differential interference contrast microscopy [3]. IFT polypeptides are found in all ciliated organisms and are highly conserved in the evolution, ranging from* C. elegans* to primates [4, 5]. The transport of IFT particles is powered by motors that include the heterotrimeric kinesin for anterograde movement [4, 6] and the cytoplasmic dynein for retrograde movement [7, 8]. Based on directionality of IFT's movement along the axoneme, IFT particles are organized into two complexes: IFT complex A and IFT complex B. In vertebrate at least six proteins (IFT43, IFTA-1, IFT122, IFT139, IFT140, and IFT144) and ten different proteins (IFT20, 27, 46, 52, 57, 74, 80, 81, 88, and 172) are in complex A and complex B, respectively [9]. IFT complex A is responsible for retrograde transport and complex B organizes anterograde transport towards the microtubule plus-ends of the ciliary tip. The bidirectional movement of IFT particles is continuous without reversing. Although the anterograde movement is slower than the retrograde movement, there is no accumulation of IFT particle in the distal tip of the cilium, suggesting cargo loading and release, IFT particle turn-around, and motor exchange are well regulated at the base and tip of cilia [10]. Most of these cargoes are derived from the Golgi body vesicles, which could explain why cilia usually form apically to the trans-Golgi network. IFT is essential for assembling all eukaryotic cilia and flagella, and defects in IFT can cause a variety of diseases and abnormal developments.

### 1.3. Photoreceptors

Photoreceptors are the photosensitive cells of the retina responsible for converting incident light into electric signals that are transmitted to the brain via the optic nerves and are interpreted by the nervous system. Rods and cones are the two types of photoreceptors, classified according to their shape and function. The human retina contains over 120 million rods and 6 million cones. Rods and cones are unevenly distributed across the retina. Some organisms, such as mouse, also have a rod dominant retina; others, such as zebrafish and squirrel, have a cone dominant retina. In humans, the central region of the retina is the macula. The central part of the macula is referred to as the “fovea,” which only contains cones and is the region showing highest visual acuity [11]. Rods are responsible for vision at low light levels (scotopic or night vision) and are very sensitive even under dim conditions. Cones are responsible for colour or day vision (photopic vision). They are much less sensitive than rods and generate signals at higher levels of light.

**Figure 1:**
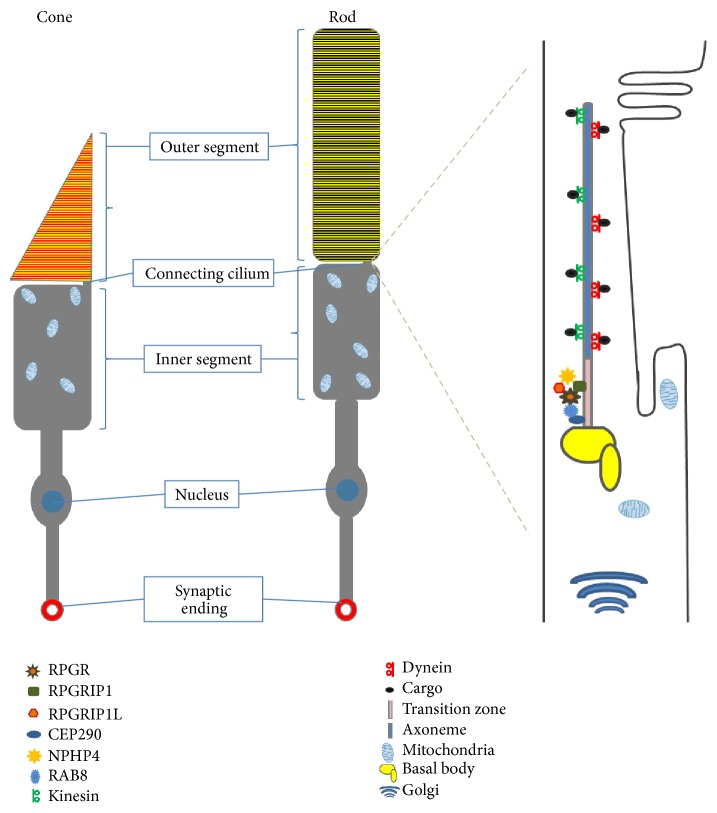
The photoreceptor primary cilia. The distinct compartments of both rod and cone cells (left side). Protein trafficking in photoreceptor connecting cilium through intraflagellar transport (IFT) is carried out by motor proteins, kinesin and dynein (right side). CEP290, centrosomal protein 290 kDa; NPHP4, nephronophthisis 4; RPGR, retinitis pigmentosa GTPase regulator; RPGRIP1, RPGR interacting protein 1; RPGRIP1L, RPGR interacting protein 1-like (right side).

Photoreceptors consist of four distinct components: a synaptic terminal, an outer segment (OS), an inner segment (IS), and a connecting cilium (CC) that connects the IS and OS (Figure 1). The OS is a highly modified primary cilium that contains numerous light sensitive stacks. These stacked lamellae are actual sites of photo transduction. The CC is a structural homologue to the transition zone of cilia [12]. Structurally rods and cones share a similar architecture. However, there is a difference in the way their outer segments are built. In rods, there are large numbers of membrane discs stacked on top of each other unconnected to the ciliary membrane, while the discs in cones are invaginations of the plasma membrane. The inner segment contains cellular organelle such as mitochondria, endoplasmic reticulum, and Golgi body so that they do not interfere with the outer segment biochemical reactions. The visual pigment in both rods and cones consists of opsin proteins. Rods are able to operate in dim light using only one type of opsin, rhodopsin, whereas cones contain several types of opsins thereby detecting different colours. The human eye contains three different types of cones for perception of different colours: blue cones (short wavelength or S cones), green cones (middle wavelength or M cones), and red cones (long wavelength or L cones) which allow us to respond to different wavelengths of light [13]. Many animals including birds, reptiles, and fish have four different types of cones. Cones have a longer lifespan than rods and do not undergo circadian phagocytosis by RPE cells [14]. The molecular processes like IFT or vesicle trafficking observed in primary cilia are also conserved in photoreceptor outer segments (Figure 1).

## 2. Ciliopathies 

The cilium proteome contains hundreds of different proteins involved in cilia protein trafficking, structure, and signal transductions [15, 16]. Deficiency of one of these proteins may be enough to produce cilia defects, giving rise to a broad spectrum of genetic disorders termed ciliopathies. Wide ranges of extracellular signals are sensed by the cilia and then transduced into decisions required for proliferation, differentiation, polarity, development, and tissue maintenance. A broad range of signals like photosensation, thermosensation, mechanosensation, hormone sensation, and olfactory sensation are received and propagated by specific ciliary receptors [17]. So, ciliary dysfunction can manifest as a variety of clinically overlapping features including primarily retinal degeneration, kidney diseases, and brain anomalies [18].

Phenotypic manifestations of ciliopathies can range from isolated blindness or renal disease to multiorgan system disorders such as retinitis pigmentosa, Meckel-Gruber syndrome (MKS), Joubert syndrome, or Bardet-Biedl syndrome (BBS). Defects of ciliary proteins can give rise to broad range of defects, including retinitis pigmentosa, hepatic, pancreatic, and kidney cyst formation, polydactyly, situs inversus, brain malformations, encephalocele, hydrocephalus, sensory defects, and skeletal abnormalities. However, mutations in the same ciliary gene can give rise to heterogeneous clinical phenotypes with different levels of severity [19]. Conversely, mutations in distinct genes can lead to the same phenotype, for example, mutations in at least eleven genes causing nephronophthisis (NPHP) [20]. In the wide phenotypic spectrum, NPHP is considered to be the mildest, BBS is intermediate in severity, and MKS is lethal. Retinal degeneration is often observed in diverse ciliopathies.

## 3. Retinitis Pigmentosa 

**Figure 2:**
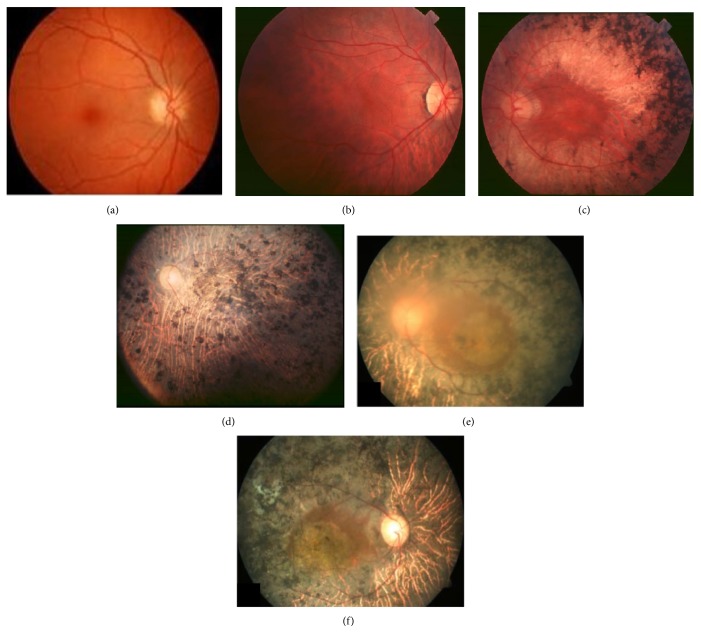
Fundus of an RP patient at different stages. (a) Image of a normal healthy eye. (b) Fundus of an RP patient at early stage. (c) Midstage of the disease showing midperipheral pigment deposits. (d) End-stage fundus showing pigment deposit present all over retina with thin retinal vessels and pale optic disc, modified from Hamel [23]. (e-f) End-stage fundus of an RP patient with an* RPGR* mutation c.852c>G (p.S284X), modified from Yang et al., 2014 [73].

Retinitis pigmentosa (RP) encompasses a group of inherited retinal degenerations that show progressive loss of photoreceptors with highly variable clinical and genetic heterogeneity. It is one of the most heterogeneous genetic disorders known in man [21], resulting from a mutation in one or more genes. It affects 1 in 4000 individuals worldwide. RP is caused by mutations in more than 50 genes, including 23 genes for autosomal dominant RP, 36 for autosomal recessive RP, and 3 for X-linked RP (XLRP) (http://www.sph.uth.tmc.edu/Retnet/). Symptoms of RP include accumulation of intraretinal pigment-like deposits, retinal vessel attenuation, and characteristic changes in electroretinogram (ERG) patterns [22]. The typical signs of early disease are night blindness and tunnel vision with progressive constriction of the visual field. The progression of typical RP can be divided into 3 stages. The early stage of the disease is within the first decade or second decade of life. The typical signs of early disease are night blindness, although at this stage visual acuity and fundus examinations appear normal; at the midstage, rod photoreceptors degeneration starts at the periphery of the retina and then progresses towards the central region during the third or fourth decade, eventually causing tunnel vision with progressive constriction of the visual field; at this stage the presence of bone spicule-shaped pigment deposits is observed in the midperiphery of the retina. At the end-stage, complete loss of peripheral vision (classical tunnel vision) is sufficiently disabling to make independent movement difficult. There is a clear deposition of pigment at different retinal regions and a clear reduction of retinal vessel thickness. At this stage cone degeneration also takes place, leading to central vision impairment (Figure 2) [23].

## 4. The Retinitis Pigmentosa GTPase Regulator (RPGR) 

**Figure 3:**
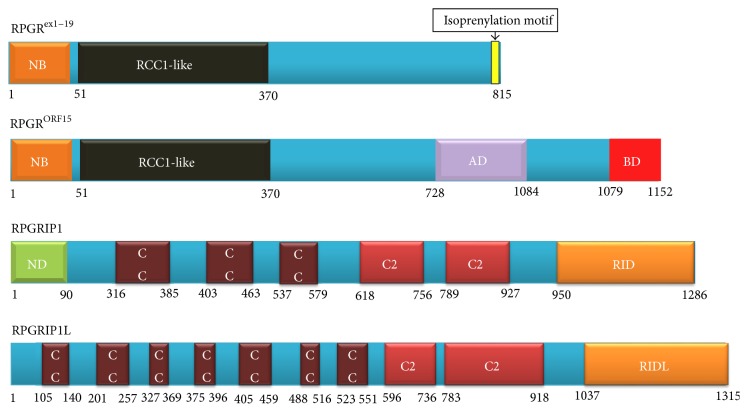
Structures of RPGR and its interacting proteins: RPGRIP1 and RPGRIP1L. The protein sequences of RPGR^ex1–19^ (NP_000319.1), RPGR^ORF15^ (NP_001030025.1), RPGRIP1 (NP_065099.3), and RPGRIP1L (NP_056087.2) were obtained from Ensembl database (http://www.ensembl.org) and domains were identified using InterPro, protein sequence analysis, classification tool (http://www.ebi.ac.uk/interpro/search/sequence-search), and details are from the literature. The isoprenylation motif (CAAX), four peptides at the N-terminal of the RPGR^ex1–19^, is marked in yellow vertical rectangle. AD, acidic domain; BD, basic domain; C2, protein kinase C conserved domain 2; CC, coiled-coil domain; NB, GTP-binding motif; ND, nuclear domain; RID, RPGR interacting domain; RIDL, RPGR interacting domain-like.

Mutations in the* RPGR* gene are the major cause of RP, accounting for more than 70% of XLRP and over 20% of nonsyndromic RP in North American families [24, 25].* RPGR* mutations are responsible for 8.8% of Japanese RP patients [26] and 4.26% of Chinese patients with cone-rod dystrophy [27]. The* RPGR* gene is located in chromosomal region Xp21.1, spanning 172 kilobases. It was first cloned in 1996 and was initially reported to consist of 19 exons [28, 29]. Later, Vervoort and colleagues identified an additional alternatively spliced C-terminal exon, called open reading frame (ORF) 15, which is highly expressed in photoreceptors [30]. The* RPGR* ^*ex1*–*19*^ transcript contains 19 exons, coding a protein with 815 amino acids with an isoprenylation motif at the C-terminus; the* RPGR* ^*ORF15*^ has 15 exons, encoding a protein with 1152 amino acids. It shares exons 1–14 with* RPGR* ^*ex1*–*19*^ plus the exon ORF15, encoding 567 amino acids with a repetitive glycine and glutamic acid-rich domain and a conserved basic C-terminal domain (Figure 3). In addition to these two major transcripts of the gene, RPGR encodes complex alternative spliced transcripts and many novel tissue-specific exons have been reported [31, 32]. All of the transcripts encode an amino (N)-terminal RCC1-like domain that is structurally similar to the RCC1 protein, a guanine nucleotide exchange factor for the small GTP-binding protein, Ran. Mutations have been reported in exon 2 to exon ORF15, where most mutations are taking place in the latter, which is regarded as a mutation hot spot [30, 33]. More than 300 mutations have been identified to cause X-linked forms of RP (Figures 2(e) and 2(f)), cone-rod, cone, and macular dystrophies, or syndromal forms of XLRP with hearing loss and primary ciliary dyskinesia [25].

**Figure 4:**
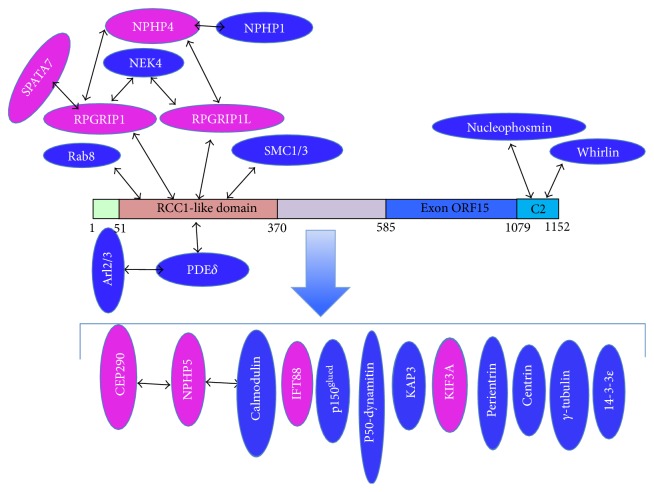
RPGR interacting protein network. Black arrows show direct interaction; the big blue arrow shows proteins in RPGR complex detected by coimmunoprecipitation. Proteins labelled in pink are implicated in retinal degeneration. The RCC1-like domain directly interacts with RPGRIP1, RPGRIP1L, SMC1/3, PDE*δ*, and Rab8. The end section of the C-terminal directly interacts with nucleophosmin and whirlin. Mutations in RPGRIP1, RPGRIP1L, CEP290, NPHP4, NPHP5, IFT88, KIF3A, and SPATA7 also cause ciliopathies.

Localization of RPGR in the retina is species dependent. RPGR is localized predominantly in the connecting cilia of mouse photoreceptors, but, in human and bovine photoreceptors, localization has been reported in the outer segments of both rod and cone cells [34, 35]. Outside of the retina, RPGR is also detected in motile cilia of airway epithelia and the centrosomes/basal bodies of cultured cells [34, 36, 37]. RPGR has been reported to interact with different proteins. The first RPGR interacting protein, the delta subunit of the rod cyclic phosphodiesterase (PDE*δ*), was identified by yeast two-hybrid screening mouse embryonic cDNA library using the RCC1-like domain [38]. Using a similar strategy, three groups independently found the RPGR interacting partner: RPGR interacting protein 1 (RPGRIP1) [39–41]. Coimmunoprecipitation and mass spectrometry analysis showed that SMC1 and SMC3 proteins interact with the RCC1-like domain [42]. The C-terminal of RPGR^ORF15^ interacts with whirlin, whose mutations cause Usher syndrome, and with nucleophosmin, which functions in cell division [36, 43]. Coimmunoprecipitation also found RPGR formed protein complexes with other ciliary proteins: IFT88, 14-3-3*ε*, *γ* tubulin, kinesin II subunit KIF3A and KAP3, dynein subunit intermediate chain, dynactin subunits: P150^Glued^ and P50-dynamitin, IQCB1 (NPHP5), and CEP290 (NPHP6) (Figure 4) [42, 44, 45]. Mutations in the* IQCB1* gene cause Senior-Loken syndrome (SLS), an inherited disorder characterized by RP and renal diseases [44]. Genetic deficiency in CEP290 is responsible for 15% of Leber congenital amaurosis (LCA) cases and has been implicated in other ciliopathies including SLS, nephronophthisis, Joubert syndrome, Bardet-Biedl syndrome (BBS), and Meckel syndrome (MKSS) [46–48]. IQCB1 physically interacts with CEP290 and both proteins involved in ciliogenesis [49].

## 5. RPGRIP1 and RPGRIP1L

Mutations in* RPGRIP1* lead to LCA, a severe retinal dystrophy causing blindness or severe visual defects at birth or in early childhood [50]. Defects in* RPGRIP1L* cause either the lethal Meckel syndrome (MKSS) or Joubert syndrome type B (JBTS), which show much broader cilia defects [51, 52]. Human* RPGRIP1* gene is located on chromosome 14q11 and encodes a protein with 1286 residues.* RPGRIP1L* gene is located on chromosome 16q12.2 and encodes a protein of 1315 residues [51]. RPGRIP1 protein has 29% amino acid identity to RPGRIP1L, which possesses similar functional domains. The N-terminals of both proteins contain coiled-coil domain with two leucine zipper motifs; the central regions contain two protein kinase C conserved region 2 (C2) domains; the C-terminal regions have an RPGR-interacting domain (Figure 3). Though the RPGR-interacting domain of RPGRIP1L is less homologous to that of RPGRIP1, the interaction with RPGR was confirmed by yeast two-hybrid and pull-down assays [53, 54]. The expression of RPGRIP1 is restricted to the retina, but RPGRIP1L is detected in retina and other tissues [35, 41, 51, 52]. Both RPGRIP1 and RPGRIP1L have similar localization to that of RPGR in retina and cultured cells [36, 51, 52]. Previous reports predicted there is a putative Ca2^+^-binding site in RPGRIP1 C-terminal C2 (C2-C) domain, but the recent crystal structure of RPGR-interacting domain of RPGRIP1 did not support the prediction [54, 55]. Both RPGRIP1 and RPGRIP1L interact with NPHP4, whose mutations result in nephronophthisis [51, 55]. Mutations in RPGRIP1, RPGRIP1L, or NPHP4 disrupted the interaction. Coene et al. used tandem affinity purification and mass spectrometry to identify that nek4 serine/threonine kinase also interacts with both RPGRIP1 and RPGRIP1L proteins [56]. More recently, SPATA7, which is responsible for LCA3 and juvenile RP, was identified as an RPGRIP1 interacting partner (Figure 4) [57]. Mislocalization of RPGRIP1 was detected in SPATA7 knockout mouse photoreceptor connecting cilium, suggesting SPATA7 is required for RPGRIP1 targeting to cilia.

## 6. The Role of RPGR and Its Interacting Proteins in Cilia Defects 

Large amounts of data from studies in cell lines and animal models suggest RPGR and interacting proteins play a critical role in cilia genesis, maintenance, and function. Knockdown of RPGR in hTERT-RPE1 cells resulted in defects in ciliogenesis with a reduced number of cilia or shortened cilia [58, 59]. Morpholino knockdown of RPGR in zebrafish embryos led to defects in retinal development, such as defective lamination of retinal cell layers and abnormal photoreceptors. Other ciliary defects in* RPGR* morphants include shorter Kupffer's vesicle cilia, a shortened body axis, kinked tail, and hydrocephaly [60, 61].* RPGR* knockout (KO) mice showed a slow retinal degeneration with photoreceptor death noted by 6 months of age [62]. Although mislocalization of cone opsins was detected as early as postnatal day 20, degenerative changes were found at age of two months. The connecting cilia of KO mice initially appeared normal and the discs of outer segments were well packed, suggesting RPGR is not necessary for the development of photoreceptors.

Knockout of* RPGRIP1* in mouse (referred to as* RPGRIP1* ^*tm1Tili*^) resulted in early retinal degeneration with most of photoreceptors degenerating by 3 months of age, resembling the phenotypes in LCA patients. The abnormal photoreceptors were apparent at the age of postnatal day 15 (P15), showing disorganized outer segments and pyknotic nuclei, a sign of ongoing cell death [63].* RPGRIP1* KO mice presented a normal structure of connecting cilia, suggesting RPGRIP1 is not needed for the connecting cilia development and maintenance. RPGR, however, lost localization to connecting cilia in* RPGRIP1* KO mice, suggesting RPGR correct localization is dependent on RPGRIP1. A recent N-ethyl-N-nitrosourea-induced RPGRIP1 null mouse model (referred to as* RPGRIP1* ^*nmf247*^) showed a more severe retinal degeneration when compared to that of* RPGRIP1* ^*tm1Tili*^ mice.* RPGRIP1* ^*tm1Tili*^ mice showed a rapid and progressive photoreceptor cell death. Outer segments were not seen in* RPGRIP1* ^*nmf247*^ mice at P7 and P14. Only 3-4 photoreceptor nuclei layers remained by P21. Rhodopsin mislocalization was detected at P12, the earliest time point examined. Other outer segment proteins, ROM1, transducin, and arrestin, were also mislocalized, but neither transducin nor arrestin mislocalized in* RPGRIP1* ^*tm1Tili*^ mice [63, 64]. The phenotype of* RPGRIP1* ^*tm1Tili*^ mice suggested that RPGRIP1 is essential for rod outer segment morphogenesis, whereas phenotypes in the* RPGRIP1* ^*nmf247*^ mice suggested RPGRIP1 is essential not only for rod outer segment disc morphogenesis but also for outer segment formation. The phenotypic difference between the two mouse mutant strains was caused by a short alternative splice variant in* RPGRIP1* ^*tm1Tili*^ mice, which may have a role in outer segment formation.

RPGRIP1L is implicated in different types of ciliopathies. Inactivation of the* RPGRIP1L* (*Ftm*) gene results in the death of mouse at midgestation and recapitulating similar malformation observed in human MKS fetues, including brain, liver, kidney, limb, and eye developmental defects [52, 65]. Morpholino knockdown of* RPGRIP1L* in zebrafish showed a broad range of ciliary defects, including shortened body axis, curved body, malformed somites, kinked notochord, abnormal tail extension, and hydrocephaly [53, 66]. In mouse cochlear hair cells, RPGRIP1L is required for planar cell polarity. In zebrafish, it is required for convergent extension and polarized positioning of motile cilia in floor plate and is also required for the stability of dishevelled protein (Dvl) at the cilium base [66].

Mislocalization of outer segment proteins has been observed in* RPGR* or* RPGRIP1* KO mice and in a patient carrying an* RPGR* mutation, suggesting RPGR and its interacting proteins are involved in protein trafficking in cilia [62–64, 67]. RPGR can regulate the ciliary trafficking of prenylated membrane-associated proteins by interacting with PDE*δ*, which is loaded with farnesylated cargo [68]. Since RPGR targeting to connecting cilia is dependent on RPGRIP1, RPGRIP1 may be involved in this process. RPGRIP1 is also essential for other ciliary protein trafficking. Mislocalization of NPHP4 and SDCCAG8, another ciliopathic protein [69], was observed in the photoreceptors of* RPGRIP1* KO mice (*RPGRIP1* ^*nmf247*^) [70]. RPGRIP1 and RPGRIP1L may function as scaffolds to recruit NEK4 to cilia, helping to maintain ciliary stability [56].

The primary cilium is a coordinating hub for different signaling pathways, such as Wnt (canonical and noncanonical), sonic hedgehog (SHH), fibroblast growth factor (FGF), Notch, platelet derived growth factor receptor *α* (PDGFR *α*), mTOR, and Hippo signaling pathways [71]. RPGR and its interacting protein may be directly or indirectly involved in these signaling pathways. Silencing of RPGR in hTERT-RPE1 caused defective cilia formation and abnormal remodelling of actin cytoskeleton [59]. The noncanonical Wnt pathway, also referred to as planar cell polarity (PCP) pathway, regulates actin cytoskeleton rearrangement by activating the small GTPase, RhoA, which plays a critical role in actin cytoskeleton dynamics. Our unpublished data suggest RPGR and its interacting protein regulate the stability of the key components of the PCP pathway. RPGRIP1L (Ftm) was shown to be involved in SHH pathway by controlling the production of Gli3 protein, which regulates SHH signaling [65]. RPGRIP1L is also involved in the PCP pathway through targeting Dvl to the cilium base and stabilizing the proteins there [66]. NPHP4, a direct interacting partner of both RPGRIP1 and RPGRIP1L, has been shown to stabilize Dvl [72]. In fact RPGRIP1L and NPHP4 coordinate to modulate dishevelled stability and control the Wnt pathway [66].

## 7. Conclusion

Mutations in the* RPGR* gene are the most common cause of RP, frequently associated with ciliopathies. RPGR directly or indirectly interacts with ciliary proteins to form one or more protein complexes. RPGR and its interacting proteins may regulate cilia genesis, maintenance, and function mainly through recruiting ciliary protein to cilia. RPGR protein complex is involved in cilia regulatory signaling pathways, but understanding the complicated RPGR-involved molecular mechanisms remains a challenge.
